# Altered Resting-State Connectivity within Executive Networks after Aneurysmal Subarachnoid Hemorrhage

**DOI:** 10.1371/journal.pone.0130483

**Published:** 2015-07-14

**Authors:** Monica Maher, Nathan W. Churchill, Airton Leonardo de Oliveira Manoel, Simon J. Graham, R. Loch Macdonald, Tom A. Schweizer

**Affiliations:** 1 Faculty of Medicine, University of Toronto, Toronto, Ontario, Canada; 2 Neuroscience Research Program, Keenan Research Centre of the Li Ka Shing Knowledge Institute, St. Michael's Hospital, Toronto, Ontario, Canada; 3 Department of Medical Biophysics, University of Toronto, Toronto, Ontario, Canada; 4 Sunnybrook Research Institute, Sunnybrook Health Sciences Centre, Toronto, Ontario, Canada; 5 The Keenan Research Centre for Biomedical Science, Li Ka Shing Knowledge Institute, St. Michael’s Hospital, Toronto, Ontario, Canada; 6 Division of Neurosurgery, St. Michael’s Hospital, Toronto, Ontario, Canada; 7 Division of Neurosurgery, Department of Neurosurgery, University of Toronto, Toronto, Ontario, Canada; Universiteit Gent, BELGIUM

## Abstract

Aneurysmal subarachnoid hemorrhage (aSAH) is associated with significant mortality rates, and most survivors experience significant cognitive deficits across multiple domains, including executive function. It is critical to determine the neural basis for executive deficits in aSAH, in order to better understand and improve patient outcomes. This study is the first examination of resting-state functional Magnetic Resonance Imaging in a group of aSAH patients, used to characterize changes in functional connectivity of the frontoparietal network. We scanned 14 aSAH patients and 14 healthy controls, and divided patients into “impaired” and “unimpaired” groups based on a composite executive function score. Impaired patients exhibited significantly lower quality of life and neuropsychological impairment relative to controls, across multiple domains. Seed-based functional connectivity analysis demonstrated that unimpaired patients were not significantly different from controls, but impaired patients had increased frontoparietal connectivity. Patients evidenced increased frontoparietal connectivity as a function of decreased executive function and decreased mood (i.e. quality of life). In addition, T1 morphometric analysis demonstrated that these changes are not attributable to local cortical atrophy among aSAH patients. These results establish significant, reliable changes in the endogenous brain dynamics of aSAH patients, that are related to cognitive and mood outcomes.

## Introduction

Aneurysmal subarachnoid hemorrhage (aSAH) is a form of stroke associated with high mortality rates and serious disability in surviving patients. Reports of mortality rates range from 32–67% [1], and approximately 33% of survivors are unable to return to work 1 year after their stroke [2]. Furthermore, over 80% of aSAH patients experience significant cognitive deficits in multiple domains, including language, memory, visuo-perceptual ability, information processing, attention and, importantly, executive function [3] [4]. Although cognitive deficits within this particular patient population are well studied, the neural correlates of these deficits are less clear. There have been studies of structural brain pathology identified at autopsy or on brain imaging after SAH, but very few have attempted to elucidate the cause and functional basis for the cognitive deficits. To our knowledge, only one study has employed functional magnetic resonance imaging (fMRI) to investigate impaired memory performance after aSAH [5], although a second study has examined the neural basis for command-following in unresponsive patients with aSAH [6]. This is an area of research that must be expanded, as elucidating the neural correlates of executive dysfunction after aSAH will strongly influence and improve understanding of patient outcomes.

In recent years, it has become increasingly common to perform resting-state fMRI (rs-fMRI) studies, where scanning is performed in the absence of explicit sensory stimuli or behavioural tasks. Rs-fMRI data are typically analyzed using functional connectivity measures, which quantify shared information between brain regions at rest, based on correlations in spontaneous, low frequency fluctuations of blood oxygen-level dependent (BOLD) signals [7]. Consistent resting-state networks have been observed across subjects and testing sessions [8] and networks show correspondence with brain regions active during cognitive, perceptual and motor tasks [9]. The use of rs-fMRI makes it possible to characterize the endogenous brain dynamics of aSAH populations; because results are not tied to a specific task paradigm, they reflect more persistent “state-like” differences in brain function associated with aSAH. Moreover, there is a growing literature consensus that rs-fMRI may prove to be a powerful diagnostic imaging tool for the assessment of stroke [10] [11].

Given that SAH is associated with deficits in higher-level cognition, an important area of study is the fronto-parietal control network, which underlies tasks requiring executive functioning [12]. This cognitive domain encompasses tasks including decision-making, working memory, language and attention [7] and the fronto-parietal control network is composed of the dorsolateral prefrontal cortex, the medial frontal gyri, superior frontal gyri, middle frontal gyri, precuneus and inferior parietal cortex [12].

Numerous fMRI studies of patients with brain lesions and healthy participants have helped to elucidate the functional roles of these network nodes. Specifically, the dorsolateral prefrontal cortex (DLPFC) and medial frontal gyri are essential for successful completion of executive tasks including the Wisconsin Card Sorting Task (WCST), Trail-Making Test (TMT), Verbal Fluency and the Attention Network Task (ANT). Patients with lesions to the right DLPFC exhibit switching deficits and impaired performance on category fluency of the Verbal Fluency Task [13], whereas patients with lesions to the left DLPFC only exhibited switching deficits [14]. This type of impairment was also seen in patients with medial frontal gyrus damage, which similarly resulted in switching deficits [14] [15]. On the WCST, patients with lesions to the DLPFC performed significantly worse overall than those with lesions elsewhere [16]. These patients also achieve significantly fewer categories, commit significantly more perseverative errors and a greater number of set losses compared with healthy controls and patients with lesions to other locations [17]. In addition, this study found that patients with lesions to the medial frontal cortex achieved significantly less categories on the WCST and committed significantly more perseverative errors–exhibiting impaired executive control. Finally, fMRI studies of healthy participants show increased activation of the DLPFC from Trail-Making Part A to Part B–consistent with this region’s posited role in executive set-shifting [18]. Currently, however, it is not known how brain activity is altered in these regions for patients with aSAH, as well as the relation between altered brain activity and stroke-related cognitive deficits.

As stated above, patients who have experienced aSAH typically exhibit cognitive deficits in domains that are linked to the frontoparietal network, although the underlying changes in brain function are currently unclear. The principal goal of this study was to establish how resting-state functional connectivity within frontal executive networks is altered after aSAH, focusing on the dorsolateral prefrontal cortex and medial frontal gyrus as key regions of interest (ROIs), and to determine what specific connectivity changes are associated with cognitive impairment. The second aim of this study was to determine whether altered functional connectivity co-varies with patient performance on several key executive tasks, along with measures of quality of life. The third goal of this study was to determine whether regional changes in functional connectivity were attributable to volumetric grey matter changes (e.g. due to cortical atrophy). This is the first study to examine functional connectivity of rs-fMRI data in aSAH patients, and provides significant, novel insights into the neural basis of post-aSAH outcomes.

## Materials and Methods

This study was undertaken with the explicit approval of the Research Ethics Board of St. Michael’s Hospital, Toronto, Canada. Both patients and controls provided written consent, which was approved by the ethics committee. Fourteen aSAH patients with aSAH (nine females, group age range: 41–67) and 14 healthy controls (nine females, group age range: 40–59) were recruited, with the groups matching in terms of age (p = 0.94) and years of education (p = 0.90). The patients were recruited according to the following inclusion criteria: 1) presentation of aSAH; 2) age range: 18–70; 3) fluent in English (as normative data for neurocognitive tests are only available in English); 4) a minimum of 8th grade education; 5) at least 3-months post-aSAH (allowing complications such as angiographic vasospasm, hydrocephalus and edema to resolve). Exclusion criteria were: 1) previous stroke; 2) previous traumatic brain injury beyond grade 2 concussion based on American Neurological Association guidelines; 3) presence of other neurological diseases (e.g., Parkinson’s disease, multiple sclerosis, epilepsy); 4) significant persisting mental illness (e.g., bipolar disorder, schizophrenia); 5) diagnosed or treated learning disabilities; 6) substance abuse unless entirely controlled for the previous 6 months; 7) hearing and visual deficits sufficiently severe to affect test performance; 8) presence of post-stroke motor deficits or seizures; and lastly 9) contraindication to MRI scanning at 3 Tesla (e.g. ferromagnetic implants or claustrophobia). Although the aneurysm clips used at St. Michael’s Hospital are safe at 3 Tesla, the latter requirement included patients who underwent coiling procedures and excluded all patients who underwent surgical clipping due to persisting concerns about imaging these patients.

### Clinical Data

Clinical data were collected for each consenting patient using Soarian Clinicals [Version 3.03, Siemens Healthcare, Germany], the online patient information database used by St. Michael’s Hospital. Variables of interest included the location (artery) of the ruptured aneurysm, laterality of the rupture (right, left or midline), size of the aneurysm, World Federation of Neurologic Surgeons (WFNS) score and presence or absence of the following complications: hydrocephalus, angiographic vasospasm, intraventricular hemorrhage and insertion of extraventricular drain. Diffusion-weighted images (DWI) and apparent diffusion coefficient (ADC) maps (obtained 2–4 days after endovascular treatment) were used to assess the number of DWI lesions. These lesions are visually identified by bright focal hyperintensities on DWI scans, with associated hypointensities on ADC maps. These lesions were determined by 2 individuals, with kappa inter-rater reliability calculated at 90%. The number of lesions recorded in these SAH patients did not differ significantly from the number seen in unruptured intracranial aneurysm (UIA) patients treated with endovascular coiling (unpublished data), therefore it is unlikely that these lesions were a result of the hemorrhage itself.

### Cognitive Analyses

Following rs-fMRI scanning (see methods below), subjects completed a cognitive testing battery including the Wisconsin Card Sorting Task (cognitive flexibility) [19], Attention Network Test (different aspects of attention) [20], Digit Span Test (working memory) [21], Digit Symbol Test (psychomotor performance) [21], Trail-Making Test parts A & B (visual attention & “set-shifting”) [22] and the Verbal Fluency task: F, A, S + Animals [23]. Questionnaires were also used to assess participants’ subjective cognitive and quality of life (QoL) complaints–the Neuropsychological Impairment Scale (NIS) [24] and the Stroke-Specific Quality of Life Scale (SS-QoL) [25].

Previous studies have found that statistically significant differences between healthy controls and aSAH patients are driven by a more severely impaired subset of patients, while another group performs equivalent to controls [26] [27], leading Al-Khindi et al. [28] to call for a more detailed reporting of cognitive deficits in groups of aSAH patients. To create a comprehensive summary of patients’ overall executive functioning, a Composite Executive Function Score (CEFS) is often generated by obtaining raw scores from individual tests, converting them to z-scores and summing them [29] [30] [31]. Patients may then be categorized as having overall executive impairment if their composite score is below zero (the group mean) and unimpaired if above zero, as per the methods used in [26] [27] [31].

Studies conducted by Possin et al. [30] incorporated tests of inhibition, set-shifting, and working memory in an executive composite score that was found to have high validity and was associated with DLPFC volumes. Prior studies used Digit Span Backwards, Trail-Making Part B, and A Brief Test of Attention within a composite neuropsychological or fronto-executive score [29] [31]. For the present study, CEFS scores were therefore computed based on the Trail-Making Test (TMT B & TMT Difference scores), Digit Span (Digit Span Forward, Digit Span Backwards & Digit Span Total scores), FAS (Correct, Errors & Switches for each letter and category), WCST (Correct, Perseverative Responses, Perseverative Errors, Trials to First Category) and ANT (Alert, Orient, Conflict, Percent Correct).

All statistical analyses of cognitive measures were performed with SPMSS Version 20, comparing between controls to the full patient group, and to impaired (CEFS < 0) and unimpaired (CEFS > 0) patient sub-groups. The non-parametric Kruskal-Wallis test was used to test for significant differences between multiple groups, and the non-parametric Mann-Whitney U test was used for two-group comparisons and post-hoc tests to identify significant variables, with p-values adjusted for multiple comparisons based on the false discovery rate (FDR) method.

### Resting-state fMRI Data and Preprocessing

Participants were scanned in a 3-Tesla Siemens Magnetom Syngo Skyra using a 20-channel head coil. During scanning, participants were instructed to remain still, with their eyes closed, and to not focus on any one concept in particular. Anatomical images were provided by T1-weighted Magnetization Prepared Rapid Acquisition Gradient Echo (MPRAGE) sequence (TR = 2000 ms, TE = 2.54 ms, TI = 1100 ms, FA = 9°; 256x192 matrix, 176 axial slices, 1.0 mm^3^ voxels), and functional data were acquired over 6.5 minutes using a T2*-weighted Echo-Planar Imaging (EPI) sequence (TR = 2000 ms, TE = 20 ms, FA = 70°; 64x64 matrix, 30 axial slices, 3.125x3.125x4.48 mm^3^ voxels). Patients recruited for the present study were scanned an average of 418 days post-stroke (range = 90–791 days), thus allowing for many clinical complications to resolve. Prior to analysis, all neuroimaging data were inspected to confirm that there was no visible coil artifact.

Neuroimaging data were preprocessed and analyzed using FSL [32] and AFNI [33] (ver. 2011/12/21.1014) software packages, with standard parameter settings. FSL’s Brain Extraction Tool (BET) was used to strip the skulls from the raw functional and anatomical data, and the first 4 EPI volumes were discarded to ensure that all subsequent data were at steady state magnetization. The remaining functional volumes were registered to the last volume in the data set using *3dvolreg* to correct for head motion. Because clinical groups often have significant motion, AFNI’s motion thresholding was applied at a fixed level across participants (afni.nimh.nih.gov/pub/dist/doc/program_help/afni_proc.py.html); no participants exhibited head motion above a threshold of 0.3mm. In addition, we compared mean average displacement of the 6 rigid-body motion parameters between patient and control groups; no significant between-group differences were identified (*p*>0.21 in all cases, Mann-Whitney tests).

Timing offsets between axial slices were also corrected using *3dTshift*. The functional data were then spatially normalized using AFNI’s “auto-Talairach” function: EPI data were matched to the T1 anatomical volume (via rigid-body transformation), the anatomical T1 images were normalized to the ICBM452 T1 atlas (www.loni.usc.edu/ICBM/Downloads/Downloads_Atlases.shtml) using 12-parameter affine warp, and functional data were warped into ICBM space using the T1-to-ICBM affine transformation matrix. Spatial smoothing was performed by *3dmerge*, with a 6mm full-width at half-maximum (FWHM) Gaussian filter, to improve signal-to-noise ratio. The *3dDetrend* program was used to regress out nuisance covariates, including 6 motion parameters and their derivatives, and Legendre polynomial regressors up to the third order; a low-pass filter was also applied with *3dBandpass* to yield band-pass frequency between 0.01–0.1 Hz, which is the primary frequency range for BOLD responses [12]. The ANATICOR algorithm was also used to estimate spatially-varying white matter signal, in order to control for physiological confounds.

### Measuring Resting-state Functional Connectivity

Functional connectivity analyses were conducted using *3dGroupInCorr* in AFNI to analyze key Regions of Interest (ROIs) in the frontoparietal network, and to identify brain regions whose BOLD time-series data were significantly correlated with the mean time-series of these key ROIs. The fronto-parietal ROI time-series were computed by selecting a seed location based on predefined coordinates in the AFNI GUI Talairach Atlas, and taking the average of all voxels within a surrounding radius of 8 mm; the selected regions included the dorsolateral prefrontal cortex (Brodmann Areas 9 and 46) and the medial frontal gyrus (Brodmann Area 6). Functional connectivity results were overlaid on the group mean T1 anatomical image, spatially smoothed with a 4.0 mm FWHM Gaussian filter to correct for small anatomic variations between subjects. The program *3dGroupInCorr* was subsequently used to generate thresholded maps of functional connectivity for a given ROI at *p* < 0.05 significance, after which *3dClustSim* was used to correct for multiple comparisons based on cluster size.

### Statistical analysis of fMRI

A Mann-Whitney U test was conducted for two-group analyses of each ROI, to test for significant differences in resting-state connectivity maps between Patient and Control groups. Additionally, comparisons were conducted for the ‘Impaired’ and ‘Unimpaired’ groups created using the CEFS results. These comparisons included: Controls versus ‘Impaired’ patients, Controls versus ‘Unimpaired’ patients and ‘Impaired’ patients versus ‘Unimpaired’ patients.

Additional functional connectivity analyses were also conducted within the aSAH patient group, to determine how connectivity covaried with two different behavioural measures. The CEFS was used as a covariate to examine how resting state networks were affected by overall executive outcomes. The ‘Mood’ measure from the SS-QoL, on which the impaired patients differed significantly from healthy controls, was also used as a resting-state covariate. Dumas et al. [34] found that reduced perfusion of the precuneus via repetitive Transcranial Magnetic Stimulation (rTMS) as associated with increased QoL in patients with depression, suggesting a key role of the precuneus in mood. Therefore, an analysis was conducted to determine whether the QoL variable ‘Mood’ exhibited a relationship with the increased connectivity between left BA46 and right precuneus. These analyses were performed using *3dGroupInCorr* in AFNI to identify clusters where changes in connectivity covaried significantly with the chosen behavioural measures at p < 0.05. We chose seed-based analysis, as we have an *a priori* hypothesis of abnormal connectivity within elements of the frontoparietal network. In this case, seed-based methods are preferred as they allow us to evaluate connections between specific regions of interest. Conversely, component-based models such as Independent Component Analysis (ICA) are more suited to exploratory whole-brain analysis (12); similarly, graph-theoretic models [35] are useful for identifying higher-order structure within large networks, which is a potential target for future studies of this population.

### Relation between Functional Connectivity and Volumetric Change

One of the challenges to interpreting changes in functional connectivity following stroke is determining the neuroanatomical basis for these changes. Recovery from stroke is associated with cortical atrophy, which may be related to cognitive outcome [36]. This includes changes proximal to the site of injury and secondary atrophy in distant regions [37], although the basis for the latter changes are not fully understood [38]. Voxel-based morphometry was conducted using FSL’s *fslvbm* protocol (fsl.fmrib.ox.ac.uk/fsl/fslwiki/FSLVBM/UserGuide), in order to determine whether regions of altered functional connectivity are driven by focal changes in grey matter density. The masked T1 volumes were segmented into probabilistic atlases of grey matter (GM), white matter and cerebrospinal fluid using *fast*, GM maps were non-linearly registered to an ICBM-152 template using *fnirt*, and the average map was computed to produce a study-specific template. The GM volumes were then registered to the group template using *fnirt*, and each voxel was rescaled by the Jacobian of the warp field to adjust for nonlinear contraction/expansion of tissue during registration. Afterwards, images were smoothed with an isotropic 3D Gaussian kernel (3mm FWHM) to improve detection power.

Maps of mean grey matter volumetric change were then computed for (1) Patients relative to Controls, and (2) impaired patients relative to unimpaired patients. The empirical p-values were then obtained for each voxel, by performing Bootstrap resampling on subjects (1000 iterations), and computing the fraction of resamples where mean change is greater than (or less than) zero. After correcting for multiple comparisons by thresholding maps at FDR = .05, regions of significant volumetric change were compared to functional connectivity maps, to determine whether these regions exhibited spatial overlap.

## Results

### Cognitive & Quality of Life Outcomes

The patient demographics and clinical characteristics are summarized for all aSAH patients in Table 1. We also split the aSAH patients into impaired and unimpaired sub-groups based on their CEFS score (as described in the Methods, this divided them into an impaired and an unimpaired group based on their mean Z scores across multiple cognitive tests). By this method, the group was split evenly, with 7 individuals displaying significant executive impairment (“impaired patients”) and 7 individuals with spared executive function (“unimpaired patients”). Comparing demographic and clinical measures for the two groups (Table 2), only the total number of DWI lesions showed a significant difference (p = 0.037; Mann-Whitney U test). Interestingly, 3 of the 4 impaired patients had DWI lesions in the left parietal cortex, as did the 1 unimpaired patient with DWI lesions. The remaining patient’s lesions were located in the left frontal cortex.

**Table 1:** Baseline clinical characteristics for all participating aSAH patients.

Characteristic		N (%)
Rupture Location	AComm	6 (42.86)
	ICA	4 (28.57)
	MCA	3 (21.43)
	PComm	1 (7.14)
Rupture Laterality	Right	3 (21.43)
	Left	5 (35.71)
	Midline	6 (42.86)
Aneurysm Size	1–4 mm	6 (42.86)
	5–9 mm	7 (50)
	≥10 mm	1 (7.14)
WFNS Score	1	12 (86)
	2	1 (7)
	3	1 (7)
Other	Intraventricular Hemorrhage	8 (57.14)
	Angiographic Vasospasm	5 (35.71)
	Hydrocephalus	6 (42.86)
	Extraventricular Drain	5 (35.71)

**Table 2:** Clinical features of patients with and without executive impairment. Mann-Whitney U and Fisher Exact tests were conducted to determine whether group differences were significant.

Characteristic		Impaired	Unimpaired	p-value
Demographic	Mean age	50.7	52.8	0.883
	Mean years education	16	14.3	0.165
Rupture Location	AComm	2	4	
	ICA	2	2	
	MCA	3	0	
	PComm	0	1	
Rupture Laterality	Right	3	0	
	Left	3	2	
	Midline	4	2	
WFNS Score	1	7	5	0.462
	2	0	1	1
	3	0	1	1
Other	Patients w/ DWI lesions	4	1	0.266
	Total DWI lesions	15	1	0.037
	Mean days post-rupture	467	370	0.461
	Angiographic Vasospasm	3	2	1
	Hydrocephalus	2	4	0.592
	Intraventricular Hemorrhage	4	4	1
	Extraventricular Drain	1	4	0.266

**Fig 1:**
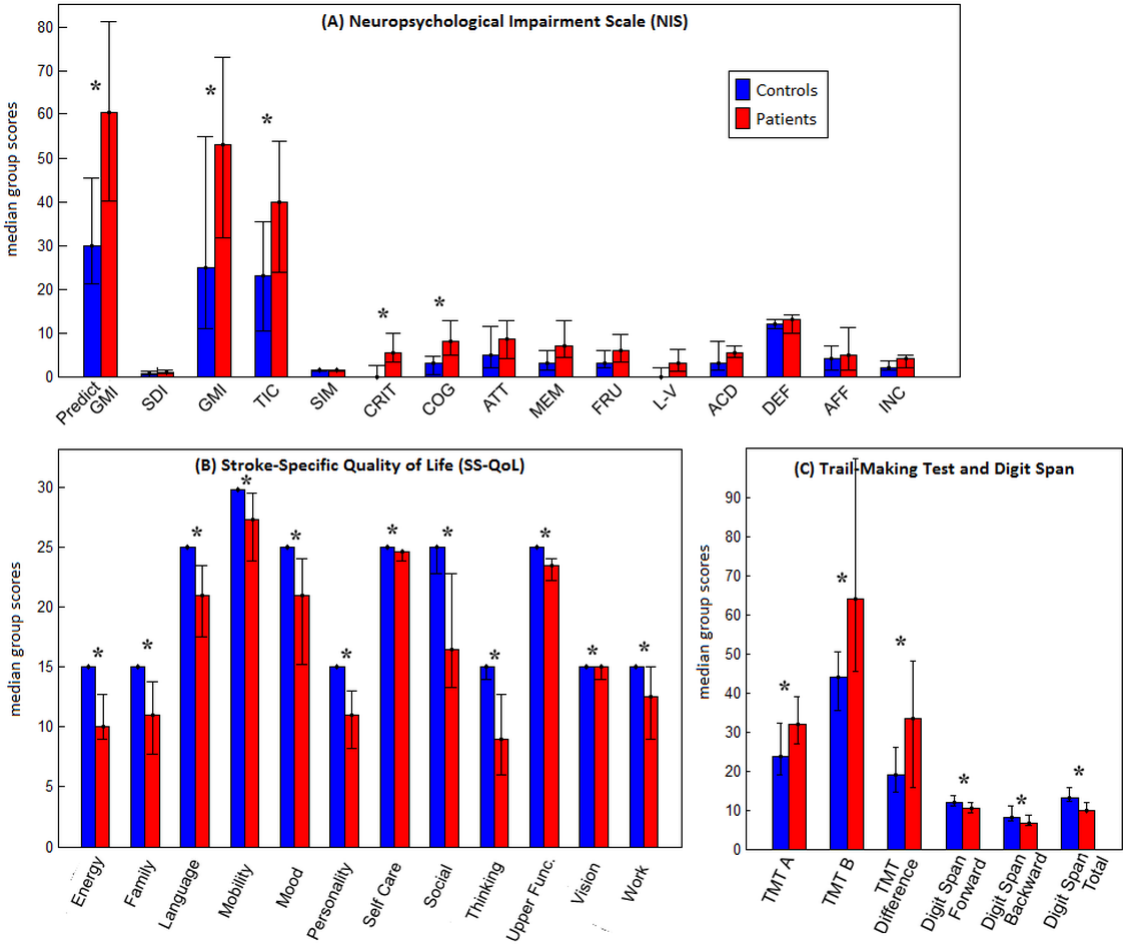
Median test scores for patient and control groups, on domains of (a) the Neuropsychological Impairment Scale (NIS), (b) the Stroke-Specific Quality of Life Scale (SS-QoL), and (c) different measures of the Trail-Making test and Digit Span. The error bars indicate the range within each group. A ‘*’ indicates significant difference between groups after False Discovery Rate correction.

Patient and control groups did not differ significantly in terms of age (p = 0.94) or years of education (p = 0.90). Patients with aSAH were impaired in terms of cognitive and raw QoL scores from the SS-QoL and NIS, and they significantly underperformed on the Trail-Making test and Digit Span test, shown in Fig 1. Results from the NIS (Fig 1A) indicate that patients had significant subjective complaints in terms of the number of cognitive symptoms and the number of symptoms associated with neurological injury (TIC and CRIT scores), and that patients’ overall rating of their own cognitive ability (COG) was significantly worse than controls. Patient results from the SS-QoL (Fig 1B) also indicated significant impairment on all 12 subscales. Importantly, we can be confident that patients’ subjective cognitive and QoL complaints as reported on the NIS were not significantly less accurate than those of controls, as their SDI (Standard Distortion Index) scores did not differ significantly (p = 0.15) from control SDI scores. In addition, patients performed significantly worse than controls on all measures of TMT and Digit Span (Fig 1C). However, the groups did not differ significantly on any of the key ANT measures, nor on any WCST variable, which was contrary to expectations. Interestingly, patients did not display any memory difficulty, as measured by the Verbal Paired Associates test (both Immediate and Delayed recall).

**Fig 2:**
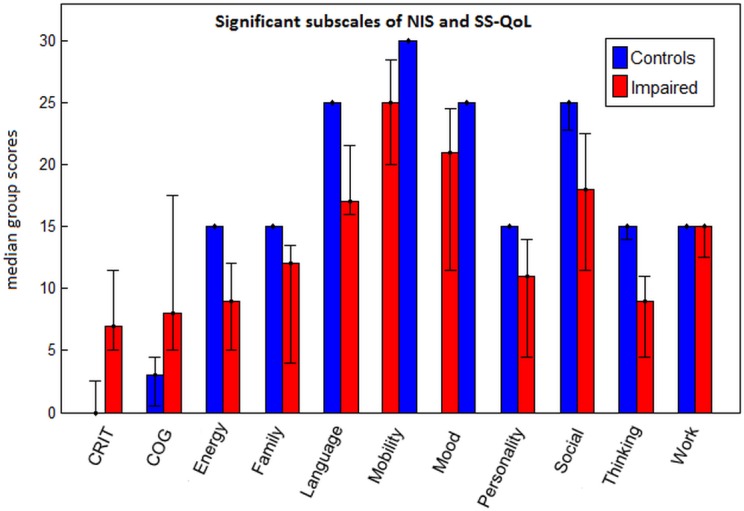
Median test scores for impaired patients and control groups on sub-scales of the Neuropsychological Impairment Scale (NIS) and Stroke-Specific Quality of Life Scale (SS-QoL), for which impaired patients scored significantly worse than controls after False Discovery Rate correction; error bars indicate the range for each group.

Next, the aSAH patient group was split based on their CEFS score into impaired and unimpaired sub-groups. By this method, the group was split evenly, with 7 individuals displaying significant executive impairment (“impaired patients”) and 7 individuals with spared executive function (“unimpaired patients”). A three-group comparison was conducted between healthy controls, impaired patients and unimpaired patients, on all cognitive tests and questionnaires, with significant between-group differences (p = 0.002; Kruskal-Wallis test). The unimpaired patients only differed significantly from controls on the “Work” sub-scale of the SS-QoL, whereas impaired patients performed significantly worse than controls on 12 subscales of both the NIS and SS-QoL together, depicted in Fig 2 (p < 0.05 for all tests; post-hoc Mann-Whitney U tests, with FDR-adjusted p-values).

**Fig 3:**
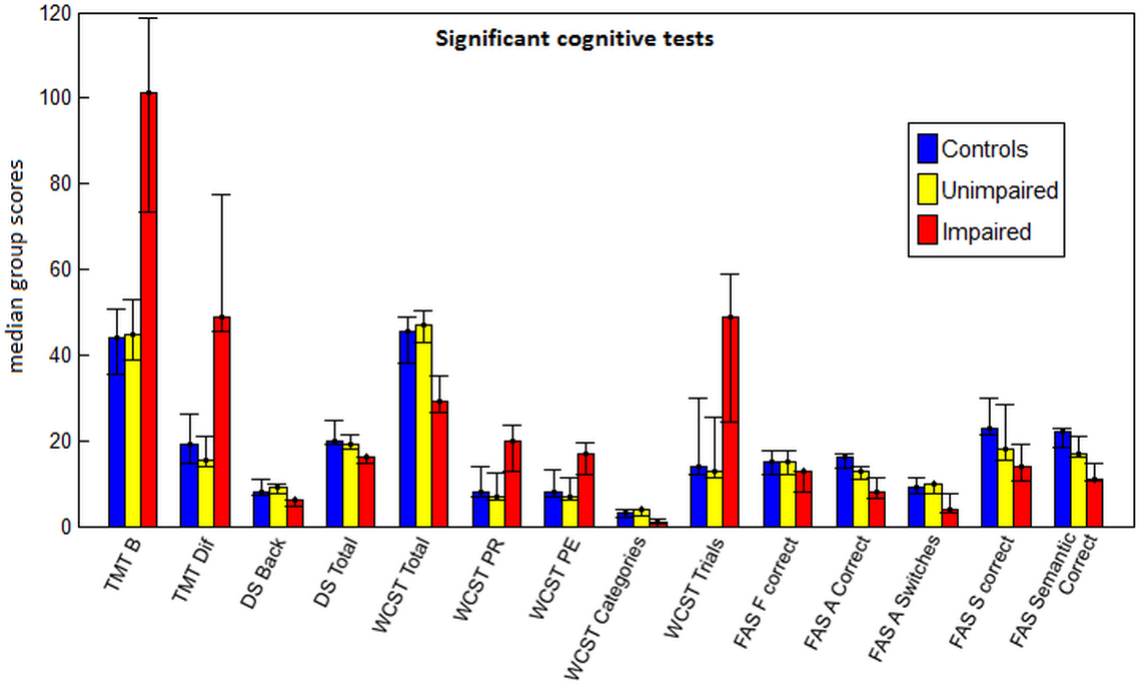
Median test scores for unimpaired patient, impaired patient and control groups, shown for cognitive tests where the impaired patients scored significantly worse than both the unimpaired patients and the healthy controls, after False Discovery Rate correction. Error bars represent the range for each group.

Furthermore, Fig 3 displays cognitive tests on which the impaired patients scored significantly poorer relative to both controls and unimpaired patients. Specifically, it took impaired patients an average of 110 seconds to complete the Trail-Making Test Part B, while unimpaired patients and controls completed the task in significantly less time (49.6 seconds and 41.6 seconds, respectively). On the WCST, patients with executive impairment made an average of 18.1 perseverative responses, nearly twice that of the unimpaired patients (average: 9.3) and controls (average: 10.3). These same patients committed an average of 16.1 perseverative errors on the WCST, compared unimpaired patients (average: 8.9) and controls (average: 9.1), respectively. Impaired patients also required more than twice as many trials (42.9 trials) for successful completion of their first category on the WCST relative to unimpaired patients (average: 16.86 trials) and controls (average: 18.46 trials). Interestingly, patients with and without executive impairment did not differ significantly on any SS-QoL or NIS measure.

### Cognitive Outcomes Related to Clinical Factors

No statistically significantly differences were observed when comparing the cognitive performance of aSAH patients with right, left or midline aneurysms (all p-values > 0.05; Kruskall Wallis test corrected for FDR) except on the number of errors on Trail-Making Part B (p = 0.008). Post-hoc Mann-Whitney U tests revealed that patients with right lateralized aneurysms committed significantly more errors on TMT B relative to left lateralized patients (p = 0.034) and midline aneurysms (p = 0.005). Additionally, there was no relationship between the number of days from the date of their aneurysm rupture to the date of their study participation and any of their cognitive scores (Spearman correlation, p > 0.05 in all cases, controlling for age and years of education). WFNS scores between impaired and unimpaired patients did not differ significantly (p = 0.46; Fisher exact test), nor did WFNS scores correlate significantly with any cognitive or QoL measure (p > 0.05; Spearman’s Rho). The number of DWI lesions significantly correlated only with the CRIT score of the NIS (p = 0.045). Finally, there were no significant differences between patients with and without hydrocephalus, angiographic vasospasm, intraventricular hemorrhage, DWI lesions or ventriculostomy.

### Between-Group Differences in Functional Connectivity

**Fig 4:**
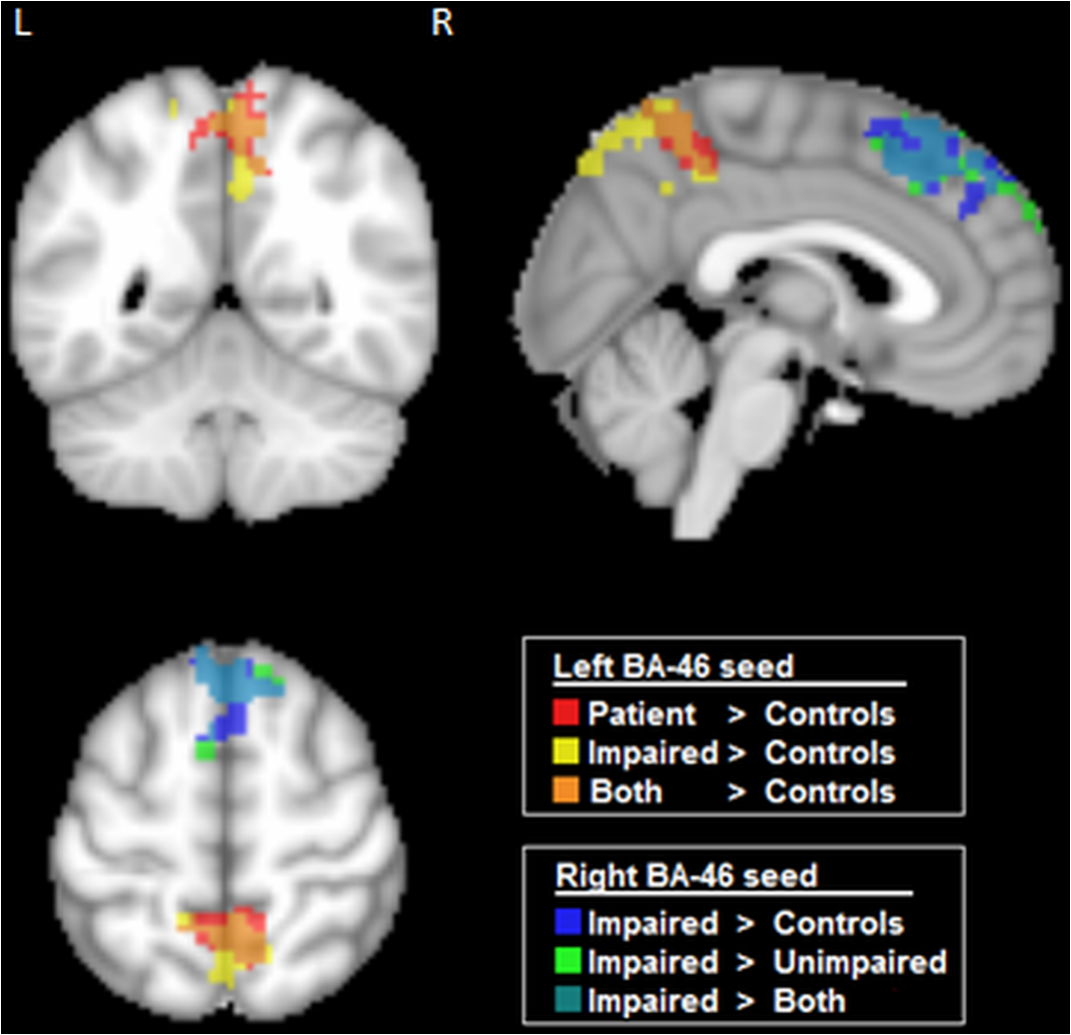
Brain regions showing significant differences in seed-based functional connectivity maps between control and patient populations. Results are shown for seeds in left and right BA46 (dorsolateral prefrontal cortex). Maps are based on pairwise comparisons between controls, aSAH patients, and patient subgroups (“impaired” and “unimpaired”, based on CEFS scores), using voxel-wise 2-sample t-tests (p < 0.05), with cluster-size correction (α = 0.05 significance level).

The functional connectivity maps of control and patient groups were compared using voxel-wise 2-sample t-tests for each of the 6 selected fronto-parietal ROIs, with cluster-size thresholding at α = 0.05. Fig 4 displays clusters with significant between-group differences in connectivity (see S1 Fig for continuous z-score scale versions of these maps); of the 6 ROIs, only left and right BA46 showed significant changes in functional connectivity maps. When comparing all aSAH patients to controls, the left BA46 seed showed a significant increase in connectivity to right precuneus (cluster size = 304 voxels; peak Z-score = 3.36; peak coordinates = (2, -49, 59)) for the patient population. After splitting patients into impaired and unimpaired groups based on CEFS, no significant differences were found between controls and unimpaired patients. Conversely, the impaired patients retained significantly higher connectivity between left BA46 and precuneus (cluster size = 278 voxels; peak Z-score = 2.53; peak coordinates = (8, -55, 59)), and also demonstrated increased connectivity between the right BA46 and the left superior frontal gyrus, relative to controls (cluster size = 437 voxels; peak Z-score = 3.15; peak coordinates = (-1, 35, 47). This cluster also included the right superior frontal gyrus and bilateral medial frontal gyri (BA8). A comparison of impaired with unimpaired patients also yielded increased connectivity between right BA46 and left superior frontal gyrus for the impaired group (cluster size = 573 voxels; peak Z-score = 2.88; peak coordinates = (-4, 32, 56)), extending into the right superior frontal gyrus and bilateral medial frontal gyri.

### Covariates of Functional Connectivity in ASAH Patients

**Fig 5:**
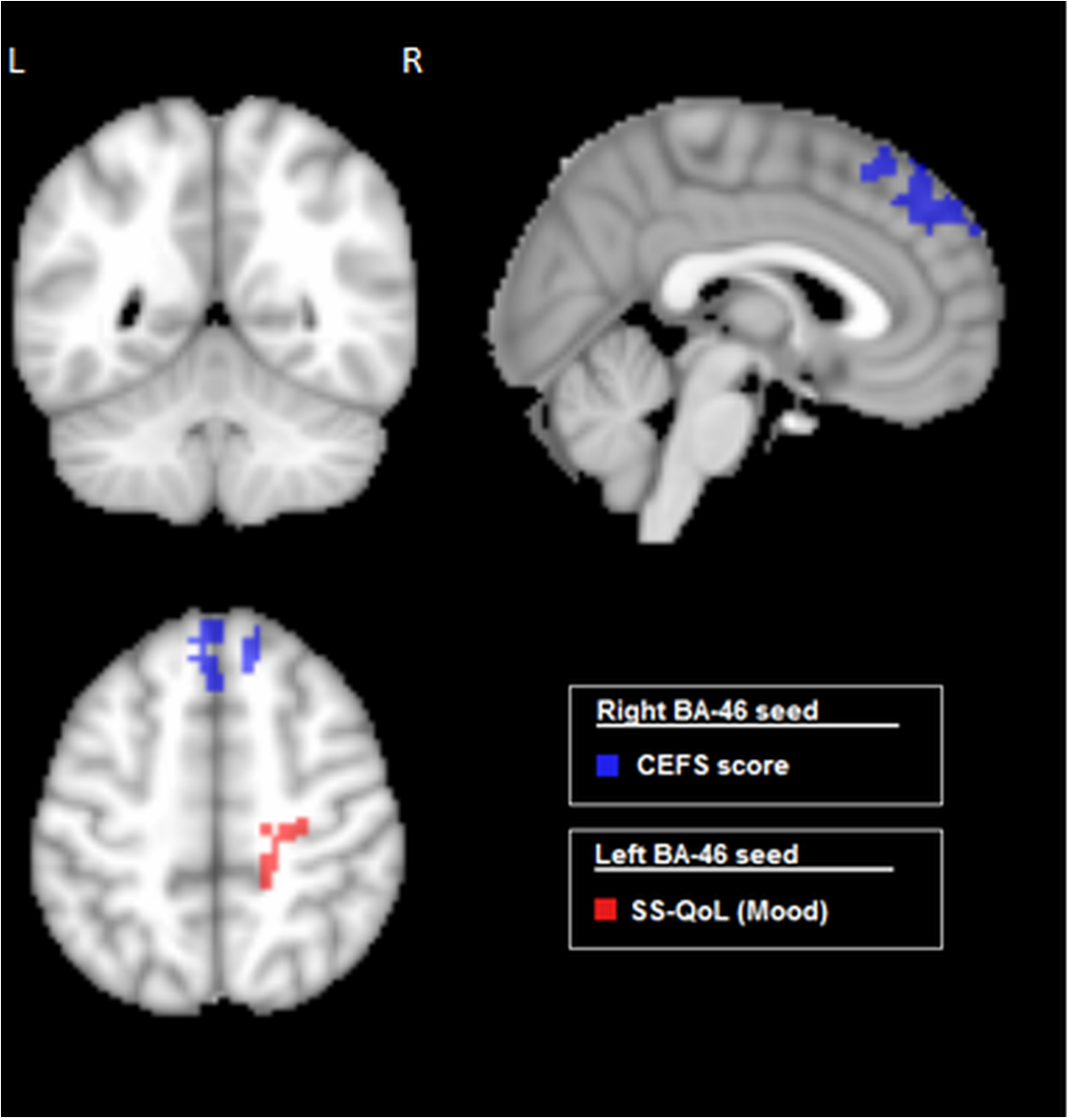
Brain regions showing significant covariation with cognitive and self-report measures, within the aSAH patient group. Results are shown for seeds in Left and Right BA46 (dorsolateral prefrontal cortex), examining the Composite Executive Function Scores (CEFS), and the “Mood” subscale of the Quality of Life (SS-QoL) scale. All significant regions exhibit negative correlation between connectivity and behavioural measures (p < 0.05), with cluster-size correction (α = 0.05 significance level).

Fig 5 shows how CEFS scores covaried with rs-fMRI connectivity maps within the patient group, to investigate whether networks are predictive of overall executive outcomes. The CEFS scores exhibited a significant inverse relationship with connectivity between right BA46 and the left superior frontal gyrus (cluster size = 326 voxels; peak Z-score = -2.043; peak coordinates = (-4, 32, 50)), extending into the left and right medial frontal gyri and right superior frontal gyrus. These results are spatially consistent with the comparison of the Impaired vs. Unimpaired groups reported in Fig 4, as expected.

Next, we determined whether the QoL variable ‘Mood’ exhibited a relationship with connectivity between left BA46 and the right precuneus in the patient group. The Mood sub-score of the SS-QoL did indeed show a significant negative covariation (p < 0.05) with connectivity of left BA46 and right precuneus (cluster size = 151 voxels; peak Z-score = -2.31; peak coordinates = (26, -39, 45)), although this cluster also extended into the right parietal lobule and precentral gyrus. This behavioural variable assesses affective disturbances such as depression, arising after stroke.

### Relation between Functional Connectivity and Volumetric Change

**Fig 6:**
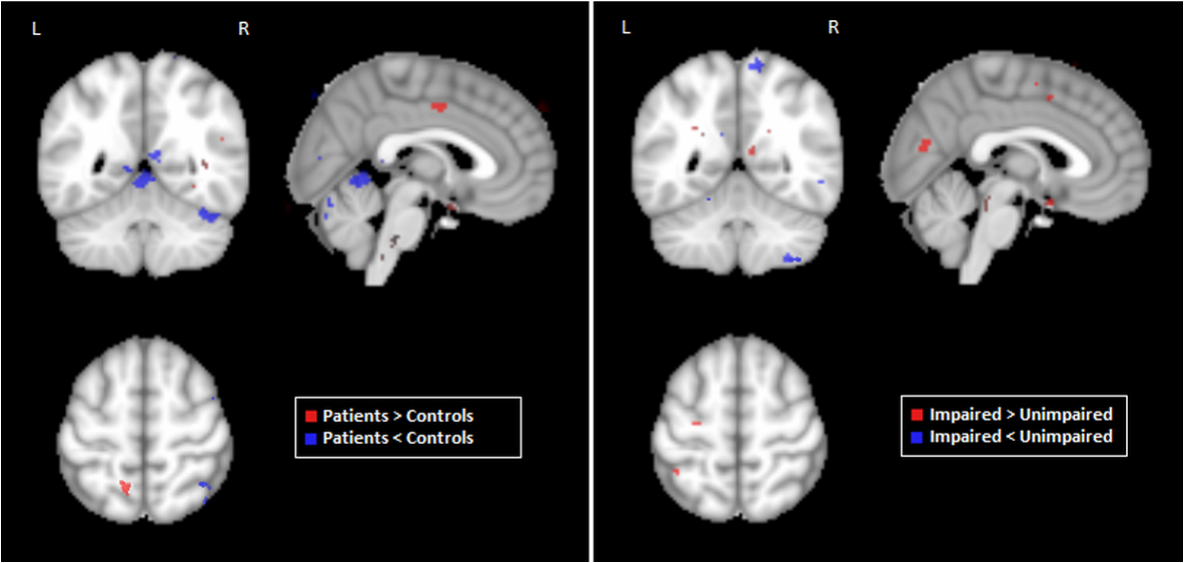
Brain regions showing significant volumetric change, when comparing (left) Patients to Controls, and (right) Impaired to Unimpaired patients. Significant regions were identified via Bootstrapped estimates of the mean difference between groups, with multiple comparison correction at False Discovery Rate 0.05.

Fig 6 depicts the results of volumetric analysis, comparing patients to controls, and Impaired to Unimpaired patients. Both comparisons demonstrate both positive and negative volumetric changes, indicating that anatomical changes due to recovery from aSAH are not limited to atrophy. Analysis of patients relative to controls shows elevated cortical volume in the left precuneus, however this has minimal overlap with the more spatially extensive functional changes (Fig 4), and the most extensive cortical changes appear in the cerebellum (negative) and middle cingulum (positive). The analysis of impaired patients relative to unimpaired shows increased volume in the left inferior parietal lobe and precentral gyrus, which are also non-overlapping with functional changes depicted in Fig 4. In this case, the most spatially extensive change appears in the primary visual cortex (positive).

## Discussion

To our knowledge, the present study is the first to examine resting-state functional connectivity in patients with aSAH. Several notable findings have emerged from this work. First, the aSAH patients show a significant increase in functional connectivity within the frontoparietal control network (left BA46 to precuneus). This network is associated with executive function, a major domain of reported cognitive deficits in aSAH patients [3] [4]. Second, the aSAH cohort was subdivided into impaired and unimpaired patient groups based on a composite executive function score (CEFS). The impaired group exhibited additional increased connectivity between right BA46 and superior frontal gyrus, relative to both control and unimpaired groups, whereas unimpaired and control groups showed no significant differences. Third, changes in functional connectivity are not associated with regional volumetric changes (e.g. due to cortical atrophy). These data provide the first evidence that cognitive impairments due to aSAH can be characterized by alterations in functional connectivity, distinct from overall differences in patients and normal control groups. Moreover, it highlights the importance of characterizing the neural correlates of behavioural heterogeneity within clinical populations.

Within the aSAH group, the CEFS scores were also shown to have a significant negative relationship with connectivity between BA46 and superior frontal gyrus, reinforcing the association between this functional connection and cognitive performance. The impaired and unimpaired groups also differed significantly on number of DWI lesions, suggesting a potential relationship between these anatomical and functional brain alterations; this is discussed in further detail below.

Increased connectivity to the precuneus was observed in the overall aSAH patient group relative to controls, as well as between impaired patients and controls. The precuneus tends to increase its activity at rest, or when an individual engages in self-reflective thought processes [39] [40], and has been identified as a major hub of the default mode network. Altered BOLD activity in the precuneus has been previously associated with depression and reduced QoL [41] [34], making our findings of connectivity relating to mood highly relevant, particularly in terms of future protocols for identifying and treating depression. Moreover, this literature supports the importance of the precuneus in characterizing healthy vs. patient fMRI data. Dumas et al. [34] applied low-frequency repetitive transcranial magnetic stimulation (rTMS) to suppress excitability in the precuneus of patients diagnosed with depression, showing that decreased perfusion of the precuneus after rTMS (assessed via SPECT) was correlated with increased patient quality of life. This raises the intriguing possibility that combined fMRI and rTMS may be worth investigating as a therapy for aSAH patients who have deficits of executive function

Depression, QoL and cognitive ability (particularly executive functioning) have been linked in studies of various neurological and psychological conditions. These studies have reiterated the importance of treating co-morbid depression in order to make any effective gain in executive functioning, which subsequently improves patients’ quality of life [42] [43] [44]. Chung et al. [45] report that approximately 75% of stroke survivors exhibit executive impairments, and that gains in this cognitive domain correlated with an improved ability to complete activities of daily living, making it essential to improve treatment for these deficits. A recent study [41] also determined that abnormal activity in the precuneus (as seen in depression, anxiety and Alzheimer disease, for example) could be altered to reduce patients’ psychopathology and in turn, improve their executive abilities. Studies investigating effective depression treatments report that the use of anti-depressants in individuals with both depression and executive dysfunction also improves overall executive ability in addition to successful management of mood [43] [46].

The number of DWI lesions was an important variable identified in the present study, as they were significantly more prevalent in the impaired subgroup of aSAH patients. The association between neurological outcomes and lesions identified from DWI/ADC scans has been well-studied [47] [48] [49]. Hadeishi et al. [48] found that patients with WFNS 1 or 2 aSAH (both clipped and coiled) did not have any identifiable lesions on DWI, however patients with WFNS 4 or 5 aSAH had numerous lesions. In the present study all patients had good neurological outcomes as measured by the WFNS scale, however it is likely that the lesions seen by Hadeishi et al. were not attributable to clipping or endovascular coiling. Frontera et al. [47] investigated ischemic lesions visualized from DWI/ADC imaging, but excluded lesions that were diagnosed as being due to endovascular and/or angiographic procedures. In a subset of cases, these lesions were associated with death (21%) or higher degree of disability (53%). The present study provides new data on DWI, showing a correlation between DWI lesions and both neurological and cognitive outcomes, as well as how these lesions may relate to abnormal neural activity after SAH. There is also evidence that patient outcome is dependent on lesion location [50]. In the current study, three of the four impaired aSAH patients with catheter-induced DWI lesions had left parietal lesions. This suggests a potential correspondence between functional changes and lesion location, although further research is required to definitively establish this relationship.

One crucial question that arises from the results of this study is why these particular resting-state connections are *increased* in the impaired aSAH patients, given that these patients perform more poorly in cognitive assessments. One interpretation is that in resting-state fMRI any observed increases or decreases relative to a normal control population merely represent deviations from the norm, as suggested in [51]. In this view, deviations are indicative of impairments, and the directionality of the change does not reflect how that region may react when it is actively engaged during tasks. Another possible explanation is that increased connectivity reflects a compensatory mechanism, attempting to offset a particular region that is not properly engaged and/or activated while completing tasks. Delnooz et al. [51] also suggest that excessive connectivity at rest causes performance deficits when these networks are recruited for cognitive tasks. Perhaps because the connection between the DLPFC and SFG is aberrant at rest, it cannot increase beyond that level when engaged to complete a task and performance is subsequently impaired. A longitudinal study is required to explore this possibility further, however. In what is currently the only fMRI study of aSAH patients, Ellmore et al. [5] found that during a working memory task these patients had increased BOLD signal throughout the entire brain (including the frontal lobes). This result might reflect an attempt to increase executive control to compensate for deficits in executive ability.

The current study had a few limitations. The number of patients studied is small; although we found significant alterations in resting state networks, this does somewhat limit our ability to characterize heterogeneity within the patient population. In addition, the current study was retrospective and limited to a single time-point 3 months post-aSAH. Prospective data collection, capturing information at multiple stages of aSAH recovery, may be required to further our understanding how resting-state changes are related to clinical, cognitive, and QoL outcomes. Although we identify robust changes in functional connectivity, BOLD fMRI is a composite measure reflecting neural metabolism, blood flow and basal physiology. This makes it difficult to establish the exact biological basis of the observed functional connectivity changes.

In the current paper, we employed voxel-based morphometry to demonstrate that functional connectivity changes are not spatially overlapped with regional cortical atrophy. Future studies examining aSAH with multiple different modalities, such as BOLD fMRI, diffusion tensor imaging (DTI), arterial spin labelling, and measures of electrophysiology (EEG and MEG) would help to clarify the biological mechanisms of these changes. For example, a recent multimodal imaging study of individuals suffering from post-concussion syndrome found that poorer cognitive performance, particularly on attention-related tasks, correlated with increased activity in the anterior cingulate but reduced structural integrity of the corpus callosum [52]. This allowed the authors to infer the compensatory mechanisms involved in attention and working memory deficits within this specific population, as well as potential processes by which this occurs.

In conclusion, aSAH patients with executive impairments exhibited abnormally increased resting-state connectivity from the dorsolateral prefrontal cortex (BA46), to the precuneus and superior frontal gyrus. Furthermore, the altered connectivity seen in these regions covaried significantly with patients’ quality of life scores and their executive scores, respectively–suggesting that this may be a neuroimaging marker for identifying aSAH patients with executive dysfunction. In future, resting-state fMRI may be used as a rapid, informative diagnostic tool to better target patients for rehabilitation of executive impairments, as well as to identify potentially disturbed mood, given previous literature that has shown that improvement of executive functioning with cognitive rehabilitation is most effective after resolution of co-morbid depression. In conclusion, the use of resting-state fMRI enables increased understanding of structural and functional alterations occurring as a result of brain injury sustained from aSAH, something that is critical to developing improved rehabilitation and better long-term patient outcomes.

## Supporting Information

S1 Fig Brain regions showing significant differences in seed-based functional connectivity maps between control and patient populations, plotted separately on a continuous z-score scale from z = 1.96 (*p* = 0.05) to the peak z-score for each map. Results are shown for seeds in left and right BA46 (dorsolateral prefrontal cortex). Maps are based on pairwise comparisons between controls, aSAH patients, and patient subgroups (“impaired” and “unimpaired”, based on CEFS scores), using voxel-wise 2-sample t-tests (p < 0.05), with cluster-size correction (α = 0.05 significance level).
(TIF)
