# Supplementary figures and images for: Altered Resting-State Connectivity within Executive Networks after Aneurysmal Subarachnoid Hemorrhage
Source: PLoS One. 2015 Jul 14;10(7):e0130483. doi: 10.1371/journal.pone.0130483 (PMC4501762; doi:10.1371/journal.pone.0130483)

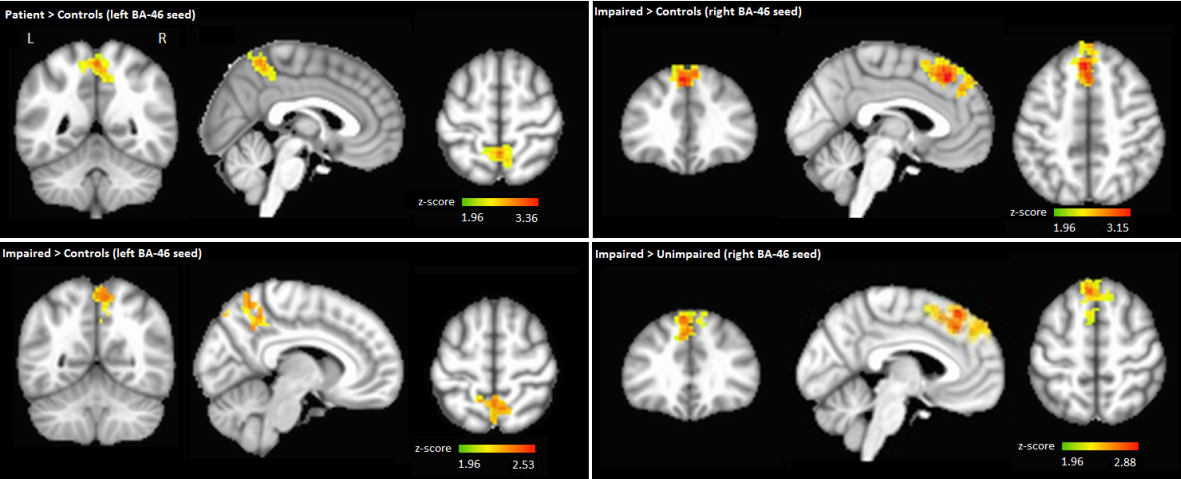

Supplement: S1 Fig — Results are shown for seeds in left and right BA46 (dorsolateral prefrontal cortex). Maps are based on pairwise comparisons between controls, aSAH patients, and patient subgroups (“impaired” and “unimpaired”, based on CEFS scores), using voxel-wise 2-sample t-tests (p < 0.05), with cluster-size correction (α = 0.05 significance level). (TIF) [file pone.0130483.s001.tif]
